# Roles of Primary Cilia in the Developing Brain

**DOI:** 10.3389/fncel.2019.00218

**Published:** 2019-05-14

**Authors:** Sang Min Park, Hee Jin Jang, Jeong Ho Lee

**Affiliations:** ^1^Biomedical Science and Engineering Interdisciplinary Program, Korea Advanced Institute of Science and Technology, Daejeon, South Korea; ^2^Graduate School of Medical Science and Engineering, Korea Advanced Institute of Science and Technology, Daejeon, South Korea

**Keywords:** primary cilia, Wnt, MTOR, autophagy, ciliopathy, FMCD

## Abstract

Essential to development, primary cilia are microtubule-based cellular organelles that protrude from the surface of cells. Acting as cellular antenna, primary cilia play central roles in transducing or regulating several signaling pathways, including Sonic hedgehog (Shh) and Wnt signaling. Defects in primary cilia contribute to a group of syndromic disorders known as “ciliopathies” and can adversely affect development of the brain and other essential organs, including the kidneys, eyes, and liver. The molecular mechanisms of how defective primary cilia contribute to neurological defects, however, remain poorly understood. In this mini review, we summarize recent advances in understanding of the interactions between primary cilia and signaling pathways essential to cellular homeostasis and brain development.

## Introduction

**FIGURE 1:**
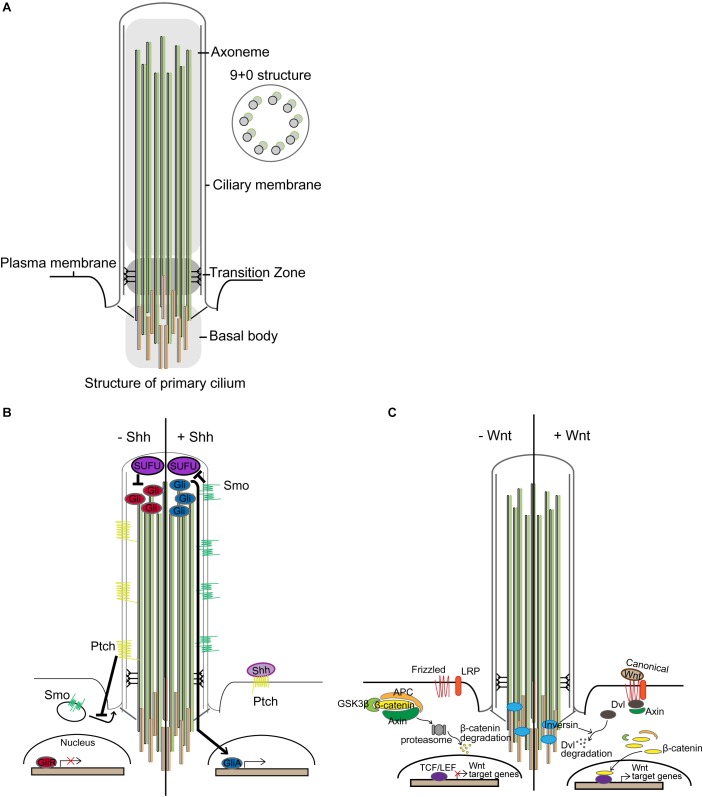
Primary cilia and signal transduction. **(A)** Structure of primary cilium. Microtubules extend from the centriole constituting the basal body and form the axoneme. The surrounding membrane is called the ciliary membrane, distinct from other membranes. Unlike motile cilium with a 9+2 structure, primary cilium has a 9+0 structure. Only ciliary proteins are allowed to access the cilium, and the transition zone performs the filtering function. **(B)** In the absence of Shh, Patched (Ptch) inhibits the translocation of Smoothened (Smo), a membrane G protein-coupled receptor (GPCR)-like protein. Suppressor of Fused (SUFU) in the tip of the cilium represses Gli, causing Gli to be present in an inactive form. In the presence of Shh, Shh binds to Ptch, making it no longer able to suppress Smo. Smo, relieved from suppression, translocates into the cilium and represses SUFU. Gli is then converted to its active form and translocates from the cilium to the nucleus and transcribes target genes. **(C)** Primary cilia in Wnt signaling. In the absence of Wnt ligand, the β-catenin destruction complex, comprising glycogen synthase kinase 3β (GSK3β), adenomatous polyposis coli (APC), and Axin, phosphorylates β-catenin, leading to its proteasomal degradation, which occurs near the basal body. Wnt signaling is activated by binding extracellular Wnt ligand to the membrane-bounded receptor family, frizzled and low-density lipoprotein receptor-related protein (LRP). Then, the β-catenin destruction complex is destabilized by anchoring Axin to the plasma membrane through Dishevelled (Dvl), which leads to stabilization and nuclear localization of β-catenin for transcriptional activation of target genes under T-cell factor/lymphoid enhancer-binding factor (TCF/LEF) promoters (van Amerongen and Nusse, 2009). Inversin, for which ciliary localization has been reported, mediates proteasomal degradation of Dvl to regulate Wnt signaling.

Primary cilia are microtubule-based cellular organelles that aid in sensing and transducing environmental signals during development (Goetz and Anderson, 2010): primary cilia transduce or regulate several signaling pathways, including Sonic hedgehog (Shh) and Wnt signaling (Corbit et al., 2005; Rohatgi et al., 2007; Gerdes and Katsanis, 2008; Goetz et al., 2009). This antenna-like structure was first observed using electron microscopy at the lumen of kidney tubules in 1898 and has since been intensively studied (Zimmermann, 1898). Emanating from the apical surface of basal body, the axoneme structure of primary cilium comprises a radial array of nine microtubule doublets lacking a central pair (9+0 structure) (Figure 1A; Davenport and Yoder, 2005). At the base of primary cilium, protein entry and exit are regulated via a transition fiber that anchors the axoneme to the ciliary membrane, compartmentalizing this distinct organelle from the cytosol while remaining continuous with the plasma membrane (Gherman et al., 2006; Marshall, 2008; Reiter et al., 2012).

Leading to defective primary cilia, mutations in genes necessary for ciliogenesis and ciliary structure and function are known to cause a group of human genetic disorders described as “ciliopathies.” To date, 187 mutated genes in 35 known ciliopathies and 241 ciliopathy-associated genes essential to ciliary structure and function that could potentially be causative for ciliopathies have been documented (Reiter and Leroux, 2017). These ciliopathies affect the body’s essential organs, including the kidneys, eyes, brain, liver, and skeleton, during development and tend to share clinical phenotypes (Hildebrandt et al., 2011): for example, as primary cilia are critical to development of the central nervous system (CNS), many ciliopathies, such as Joubert syndrome (JBTS), Meckel syndrome (MKS), and orofaciodigital syndrome (OFD), commonly exhibit neurological defects of CNS malformation, intellectual disability, ataxia, and retina dystrophy (Valente et al., 2014). Here, we summarize our recent understanding of the interactions between primary cilia and several signaling pathways essential for cellular homeostasis and for brain development.

## Signaling Pathways and Biological Processes Mediated by Primary Cilia

### Shh Signaling

Sonic hedgehog signal transduction is accomplished through binding of Shh to the transmembrane receptor Patched (Ptch). In the absence of Shh, Ptch inhibits the ciliary localization of Smoothened (Smo), a membrane G protein-coupled receptor (GPCR)-like protein. When present, Shh binds to Ptch to alleviate the suppression of Smo, which, in turn, translocates to the primary cilium to repress Suppressor of Fused (SUFU) (Rohatgi et al., 2007; Tukachinsky et al., 2010; Sasai and Briscoe, 2012). This initiates the activation of Gli transcriptional factors (Figure 1B; Alcedo et al., 1996): Gli regulates the transcription of target genes that regulate the Shh signaling (e.g., Ptch1, Gli1), proliferation (e.g., cyclin D1, MYC), and apoptosis (e.g., Bcl-2) (Scales and de Sauvage, 2009). Shh, Ptch, Smo, and Gli are expressed in the developing and adult brain (Echelard et al., 1993; Ekker et al., 1995).

Sonic hedgehog signaling is critical to spatial patterning of the neuroepithelium, cellular identity in CNS, axonal guidance, wiring of the neural network, and neuronal activity. As Shh signaling-related proteins are located within primary cilium, Shh signaling is not properly achieved in mice with defective cilia, resulting in several defects in brain development such as defective neural patterning, cerebellar hypoplasia, and defective hippocampal neurogenesis (reviewed in Ferent and Traiffort, 2015). For example, mice with mutation in *Kinesin family member 3A (Kif3a)*, which is important for ciliogenesis, show reduced Gli1 expression, thereby leading to defects in hippocampal neurogenesis and formation of neural stem cells (Han et al., 2008). *Stumpy* mutant mice exhibit defective ciliogenesis leading to prenatal hydrocephalus and severe polycystic kidney disease. The *Stumpy* gene, located on mouse chromosome 7, encodes B9 protein domain 2 (B9D2), which is localized with basal bodies along ciliary axonemes and appears to play a role in ciliogenesis in association with intraflagellar transport proteins (IFTs) (Town et al., 2008). Conditional *Stumpy* mutant mice (driven by Nestin-Cre) have been found to show abrogated Shh signaling and Gli processing in the hippocampus (Breunig et al., 2008).

### Wnt Signaling

Wnt signaling is another developmental signaling pathway mediated by primary cilia (Figure 1C; May-Simera and Kelley, 2012). Wnt signaling can be divided into (1) β-catenin dependent (canonical) and (2) β-catenin independent (non-canonical) signaling (reviewed in Berbari et al., 2009; Oh and Katsanis, 2013). Several studies have shown that perturbation of ciliary genes aberrantly activates canonical Wnt signaling and disrupts non-canonical Wnt signaling (Lin et al., 2003; Cano et al., 2004; Ross et al., 2005; Simons et al., 2005; Gerdes et al., 2007; Corbit et al., 2008). In particular, Inversin, which is localized in cilium, mediates the proteasomal degradation of Dishevelled (Dvl) to regulate both canonical and non-canonical Wnt signaling (Simons et al., 2005). Perturbations in several ciliopathy-related genes have been found to elicit de-regulated canonical Wnt signaling in fibroblasts and in the forebrain (Willaredt et al., 2008; Abdelhamed et al., 2013; Wheway et al., 2013). Also, ciliary ablation in adult-born dentate granule cells in the hippocampus has been shown to aberrantly activate canonical Wnt signaling, concomitant with severe defects in dendritic refinement and synapse formation (Kumamoto et al., 2012). Additionally, researchers have recently demonstrated that defective neuronal ciliogenesis caused by hyperactivating mutation in *MTOR* elicits abnormal activation of canonical Wnt signaling and inactivation of non-canonical Wnt signaling, resulting in defective neuronal migration due to disrupted neuronal polarization (Park et al., 2018). Notwithstanding, several studies have reported that ciliary dysfunction does not affect Wnt signaling, as evidenced by constant activity of Wnt signaling reporter and expression of Wnt target genes (Huang and Schier, 2009; Ocbina et al., 2009). Interestingly, however, deregulated canonical Wnt signaling via deletion of adenomatous polyposis coli (APC) was found to cause a loss of primary cilia in association with radial progenitor malformation in the neocortex (Nakagawa et al., 2017). In these regards, the interaction between primary cilia and Wnt signaling may be context-dependent, and more thorough studies will be necessary to elucidate it.

### MTOR Signaling

**FIGURE 2:**
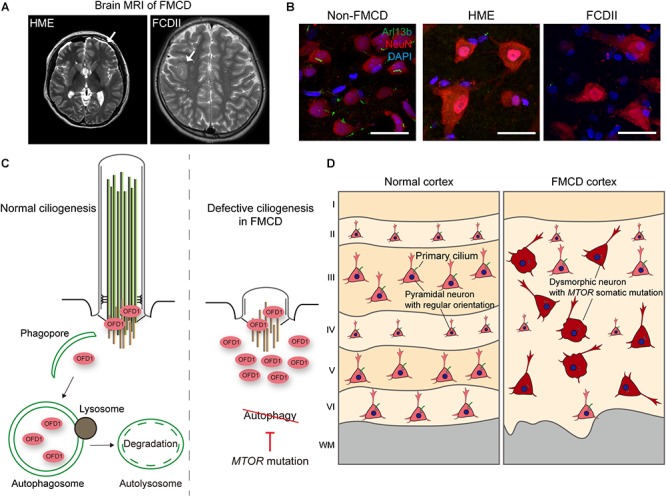
Defective ciliogenesis due to brain somatic mutations in *MTOR* accounts for cortical dyslamination in FMCDs. **(A)** Representative brain MRIs of patients with FMCDs, including hemimegalencephaly (HME) and focal cortical dysplasia (FCD) type II. Arrows indicate the affected region of the brain. Adapted from Park et al. (2018), with permission. **(B)** Immunostaining for Arl13b, a marker for primary cilia, and NeuN, a marker for neurons, with DAPI co-staining in brain tissue from FMCD patients. While primary cilium normally forms at each neuron in brain tissue from non-FMCD, FMCD patients with brain somatic mutations in *MTOR* exhibit defective neuronal ciliogenesis. Scale bars, 30 μm. Adapted from Park et al. (2018), with permission. **(C)** Autophagic degradation of the centriolar satellite pool of OFD1 induces primary ciliogenesis. However, brain somatic activating mutation in *MTOR*, which blocks autophagy, disrupts ciliogenesis in brain tissues with FMCDs. **(D)** Cortical dyslamination with ciliary defective dysmorphic neurons in the cerebral cortex of a FMCD patient. Somatic activating mutations in *MTOR*, causative for FMCDs, disrupt neuronal ciliogenesis through blockage of autophagy, resulting in cortical dyslamination.

MTOR is a serine/threonine kinase essential for protein translation, lipid synthesis, and autophagy, and plays an essential role in neural differentiation, neuronal migration, and synaptic formation, all of which are crucial to brain development. Disruption of MTOR signaling has been documented in numerous pathological conditions, including cancer, neurological disorder, and metabolic disorder (Lipton and Sahin, 2014; Saxton and Sabatini, 2017) Polycystic kidney disease (PKD) patients with inherited mutations in ciliary genes show deregulation of the MTOR signaling pathway (Shillingford et al., 2006; Wahl et al., 2006). In particular, *polycystin-1*, which is recurrently mutated in PKD, has been shown to inhibit MTOR through interaction with TSC2, a component of the TSC complex that inhibits MTOR (Shillingford et al., 2006). Studies have also identified that, by bending stimulus via fluid flow, primary cilia downregulate AMPK-MTOR signaling, which, in turn, induces autophagy to control cell size via LKB1 localized at primary cilia in the kidneys (Boehlke et al., 2010; Orhon et al., 2016). Recently, research has demonstrated that, during brain development, mice with defective cilia present abnormal increases in MTOR signaling, leading to enlarged apical domains of radial glial cells (RGCs) and subsequent dilatation of brain ventricles (Foerster et al., 2017). Conversely, in *TSC1*- or *TSC2*-null fibroblasts, TSC1 and TSC2 have been found to positively regulate ciliogenesis without using the TSC-MTOR signaling axis (i.e., rapamycin-insensitive) (Hartman et al., 2009). Nevertheless, a recent independent study which also performed with *TSC1*- or *TSC2*-null fibroblasts reported different ciliary phenotypes marked by a longer ciliary length in *TSC1*-null cells and a shorter ciliary length in *TSC2*-null cells, and there was a discrepancy between the two cell types in that only the elongated ciliary phenotype in *TSC1*-null cells could be rescued by rapamycin treatment (Rosengren et al., 2018). In focal malformations of cortical developments (FMCDs), such as hemimegalencephaly (HME) and focal cortical dysplasia (FCD), which are highly associated with intractable epilepsy and autism-spectrum disorders, brain somatic activating mutations in *MTOR* eliciting blockage of autophagy have been described as disrupting neuronal ciliogenesis in brain tissues from FMCD patients (Wegiel et al., 2010; Lim and Crino, 2013; Park et al., 2018; Figure 2A,B). Given the complex reciprocal interaction between primary cilia and MTOR signaling, further mechanism studies are yet needed.

### Autophagy

Autophagy is a cellular degradative process by which a cell gains nutrients to maintain homeostasis (Mizushima, 2007). Two independent studies have provided evidence of links between ciliogenesis and autophagy: In serum-nutrient conditions, basal autophagy degrades the ciliary protein IFT20, while OFD1 protein at centriolar satellites inhibits ciliogenesis. Upon serum-withdrawal, the centriolar satellite pool of OFD1 is degraded by inducible autophagy, while IFT20 initiates ciliary trafficking, leading to ciliogenesis (Pampliega et al., 2013; Tang et al., 2013). Cilia-mediated Shh signaling has been found to assemble several proteins needed for autophagy at the periciliary region, thus activating Shh signaling induces autophagy flux. Interestingly, one study described defects in serum-withdrawal-induced autophagy and biogenesis of autophagosome among Ift20- or Ift88-compromised cells (Pampliega et al., 2013). In line with this, others have found that treatment of Shh to cultured-hippocampal neurons upregulates several autophagy-related genes and enhances autophagy (Petralia et al., 2013).

Meanwhile, dysregulated ciliogenesis in relation to defective autophagy has been described in several diseases. Previous studies have reported suppressed autophagic flux in ciliary dysfunctional PKD mouse models and impairment of autophagosome formation in cells derived from PKD patients (Belibi et al., 2011; Zhu et al., 2017). Interestingly, Hürthle cells found in thyroid cancer were found to show defective ciliogenesis, and this defect in Hürthle cells eliciting high basal autophagic flux was restored by autophagy inhibition (Lee et al., 2016). Dysregulated ciliogenesis has also been reported in Huntington’s disease (HD), for which defective autophagy has been well recognized (Keryer et al., 2011; Kaliszewski et al., 2015). Although *Huntingtin*, which is mutated in HD, is known to regulate autophagy, it also participates in trafficking pericentriolar material 1 (PCM1) to the centrosome. When *Huntingtin* is mutated, aberrant accumulation of PCM1 occurs, which, in turn, causes dysregulated ciliogenesis (Keryer et al., 2011; Rui et al., 2015). Further studies, however, are required to elucidate the direct interaction between ciliary abnormality and defective autophagy in HD. Finally, as stated in the previous section on MTOR signaling, defective autophagy and resultant disruption of ciliogenesis have been demonstrated in FMCDs (Park et al., 2018). Brain somatic activating mutations in *MTOR*, which are causative for FMCDs, block autophagy, resulting in defective neuronal ciliogenesis due to aberrant accumulation of OFD1 (Figure 2C; Park et al., 2018). Although defective autophagy has been implicated in many neurodevelopmental and neurodegenerative disorders, much more studies are needed to elucidate the pathophysiological role of primary cilia in these disorders (Levine and Kroemer, 2008; Lee et al., 2013; Marsh and Dragich, 2018).

## Developmental Functions of Neuronal Cilia

### Patterning and Morphogenesis of the Forebrain

One of the well-established developmental functions of primary cilia is to control forebrain patterning, which is severely defected in human ciliopathies (Vogel et al., 2012). Patterning in the neural tube of the CNS occurs through progressive subdivisions along the dorsal to ventral and the rostral to caudal axes (Dessaud et al., 2008). *Cobblestone* mice (*Ift88^cbs/cbs^*), which show severe disorganization of the telencephalon, have been found to exhibit an ambiguous dorsal-ventral forebrain boundary and dorsal telencephalic-diencephalic boundary, concomitant with increased levels of full-length Gli3 (Willaredt et al., 2008). Meanwhile, mice lacking primary cilia as a result of mutation in the ciliopathy gene *Rpgrip1l* exhibited defective phenotypes associated with MKS and JBTS. In these mice, a mislocalized olfactory bulb-like structure and ambiguous dorsal-ventral patterning were observed, both of which were rescued upon introduction of constitutively active repressor form of Gli3 (Besse et al., 2011). Other mice with defective cilia through loss of *Ttc21b* (encoding retrograde Ift139) have also been shown to display defects in dorsal–ventral patterning and rostral–caudal patterning. Researchers have reported that *Ttc21b*-null mice exhibit normal anterograde transport that permits entry of Shh signaling components into cilium but partially defective retrograde transport. Overactivation of GLI2 and GLI3A in these mice could accounts for activation of Shh signaling and abnormal patterning, suggesting a periciliary role for retrograde IFT in GLI3 processing and GLI2 activity (Huangfu and Anderson, 2005; May et al., 2005; Tran et al., 2008; Stottmann et al., 2009). Crossing *Ttc21b*-null mice with mice heterozygous for a null allele of *Shh* to reduce levels of Shh ligand was found to partially rescue their defective phenotypes (Tran et al., 2008; Stottmann et al., 2009). This paradoxical effect of retrograde IFT mutants on Shh signaling (i.e., activation of Shh signaling) was well reviewed by Pal and Mukhopadhyay (2015) and Bangs and Anderson (2017). In line with the importance of primary cilia in cortical patterning, mice with mutant *Slb* (encoding anterograde Ift172) that possess very short axonemes with no visible microtubules in their cilia have also been found to exhibit a global brain-patterning defect along the dorsal–ventral and rostral–caudal axes (Gorivodsky et al., 2009). Collectively, these studies suggest the importance of Gli3 processing, which could be mediated by primary cilia, in forebrain patterning.

The establishment of polarized radial glial scaffold formation during cortical development has been shown to be regulated by Arl13b, a small GTPase enriched in primary cilia (Higginbotham et al., 2013). *Arl13b* mutant mice (*Arl13b^hnn/hnn^*), which display defective cilia, surprisingly exhibit dramatic inversion of the apicobasal polarity of the radial glial scaffold, which, in turn, changes the location of the progenitor zone to the outer part of the cortex. This defect is also observed when *Arl13b* is removed just before the formation of radial glia from neuroepithelial cells using Foxg1-driven Cre. However, *Arl13b* removal after establishment of the radial glial scaffold using Nestin- or GFAP-driven Cre has not been found to generate this intriguing phenotype, suggesting that the role of Arl13b in polarization is temporally restricted to the period during which the radial glial scaffold develops (Higginbotham et al., 2013). This reversed apicobasal polarity seen in *Arl13b* mutant mice has not been reported in mice deficient of other ciliary genes. In line with this temporally restricted role of a ciliary gene during development, a recent study performed conditional knockout (KO) of *Kif3a*, *Ift88*, and *Ttc21b* in a series of specific spatiotemporal domains. Intriguingly, the observed neurological defects, including forebrain expansion, cortical malformations, impaired olfactory bulb development, and ventriculomegaly, were significantly different across four different Cre transgenic alleles (Foxg1-Cre, Emx1-Cre, Wnt1-Cre, and AP2-Cre), indicating that the roles of cilia in cortical development are discretely spatiotemporal. For example, the deletion of *Ift88* at earlier stages of development, driven by Foxg1-Cre, was found to increase brain size; however, when deleted at later stages, driven by Emx1-Cre, there were no significant differences in brain size (Snedeker et al., 2017). Taken together, these experimental studies of perturbations of primary cilia in mice support the crucial role of primary cilia in forebrain development, which, when disrupted, lead to severe morphological malformations in the brain.

### Neuronal Migration

During brain cortical development, newly born neurons follow one of two main migratory routes to reach their final destination, known as radial and tangential migration (Hatten, 1999). The role of primary cilia in radially migrating pyramidal neurons remains unclear. In a previous study, cerebral cortical lamination was not apparently affected in *Arl13b*-conditional KO mice driven by Nex-Cre or in *Stumpy*-, *Kif3a*-, and *Ift88*-conditional KO mice driven by Nestin-Cre (Arellano et al., 2012; Higginbotham et al., 2012; Tong et al., 2014). However, a recent *in vivo* systemic study revealed that genes linked to ciliopathies affect cortical development, including neuronal radial migration, neural progenitor development, neuronal differentiation, and early neuronal connectivity (Guo et al., 2015). In this study, knockdown of 17 ciliopathy genes in the developing neocortex resulted in delayed neuronal radial migration in association with transient multipolar stage, multipolar-to-bipolar transition, and glial-guided radial migration (Guo et al., 2015). Meanwhile, others have suggested that the defective phenotypes seen in ciliary conditional KO mice driven by Nestin-Cre, the expression of which is broad and is also seen in non-RGCs at later developmental stages, may be partly attributable to non-cell-autonomous effects (Foerster et al., 2017; Chen et al., 2018). Accordingly, the cell-autonomous function of primary cilia in neural stem cells at the late developmental stage was investigated via acute knockdown of ciliary genes (*Kif3a* or *Ift88*) in the developing cortex, which resulted in defective neuronal differentiation and migration; this was accompanied by delays in neural stem cell cycle progression and failures in interkinetic nuclear migration (Chen et al., 2018). In FMCDs, the molecular mechanism of how somatic mutations in *MTOR* lead to focal cortical dyslamination appears to be related to defective neuronal ciliogenesis by *MTOR* mutation, which affects the multipolar to bipolar transition essential for neuronal radial migration (Figure 2C,D; Park et al., 2018).

Interneurons originating from the medial ganglionic eminence migrate along a tangential path in the cortical plate, either at the marginal zone or intermediate zone. These neurons change their orientation from a tangential to radial path to colonize the cortical plate and to differentiate into GABAergic neurons (Guo and Anton, 2014). Recent studies have demonstrated that disruption of primary cilia at interneurons affects the interneuronal migratory process, suggesting the importance of primary cilia in neuronal migration (Baudoin et al., 2012; Higginbotham et al., 2012). In a previous study, mice with conditional KO of *Arl13b* genes driven by Dlx5/6-Cre showed disruption of interneuron placement at *Arl13b*-deleted cortices caused by defects in interneuron migration and branching. However, when the *Arl13b* gene was disrupted in post-migratory interneurons driven by PV-Cre, there was no significant difference in the morphology of dendritic arbors during neuronal differentiation. Such defective interneuron migration may be a potential pathophysiological mechanism underlying neurological defects in JBTS with *Arl13b* mutations (Higginbotham et al., 2012). Meanwhile, a recent study showed that disruption of cilia in interneurons using Nkx2.1-Cre to ablate *Ift88* or *Kif3a* genes led to defective migration of interneurons, marked by an aberrant accumulation of interneurons at their first tangential path, not radial streams in the cortical plate, as well as abnormal positioning and density of interneurons in the postnatal cortex (Baudoin et al., 2012). Taken together, these studies emphasize that primary cilia in immature interneurons are crucial to interneuron migration in the developing cortex and the underlying mechanisms thereof (Baudoin et al., 2012). Nevertheless, the role of primary cilia in neuronal migration needs further study, especially in regards to which other cilia-mediated signaling pathways are involved. These future studies on neuronal migration should shed light on the pathophysiology of ciliopathies presenting with cognitive deficits, which may be attributable to defective neuronal migration and disrupted neural connectivity.

### Cerebellum Development

Ataxia due to cerebellar malformation is one major symptom of ciliopathies, particularly JBTS. During development of the cerebellum, Purkinje cells secrete Shh, which regulates the proliferation of cerebellar neuronal precursor (Wechsler-Reya and Scott, 1999; Haldipur et al., 2012): Shh signaling mediated by primary cilia occurs in late embryonic stages of cerebellum development, during which Shh is required for expansion of the granule neuron precursor population, but not for the subsequent differentiation of these cells (Lewis et al., 2004). Primary cilia control cerebellar morphogenesis by promoting the expansion of the granule progenitor pool. Loss of either *Ift88* or *Kif3a* causes severe cerebellar hypoplasia and inhibits the expansion of the granule cell progenitor population. Although Ift88 and Kif3a are not required for the specification and differentiation of cerebellar cell types, they affect granular cell progenitor proliferation. Researches have shown that the expression of Gli1 at the external granule cell layer in *Kif3a* mutant mice is lower than that in controls (Chizhikov et al., 2007; Spassky et al., 2008). These studies demonstrate that ablation of primary cilia impairs Shh signaling, which regulates the development of the cerebellum, particularly expansion of cerebellar progenitors. This mechanism could explain the hypoplasia of the cerebellum and cerebellar ataxia seen in patients with ciliopathies such as JBTS.

### Learning and Memory

Patients with ciliopathies commonly present with intellectual disability (ID) as a neurological deficit. Although there are not enough studies to explain the mechanism underlying ID as a result of defects in primary cilia, several studies have shown that depletion of primary cilia leads to hippocampal-dependent learning and memory deficits (Breunig et al., 2008; Han et al., 2008; Amador-Arjona et al., 2011). Hippocampal neurogenesis plays a role in fear conditioning, recognition, spatial memory, and pattern separation (Cameron and Glover, 2015). Also, Shh signaling mediated by primary cilia controls the proliferation of neural progenitor cells (NPCs). As evidence thereof, Emx1-Cre-dependent *Shh*-conditional KO mice were found to show smaller dorsal telencephalons at E18.5 and decreased proliferation and increased apoptosis of neural stem cells (NSCs) and NPCs in the dorsal pallium (Komada et al., 2008). Meanwhile, Nestin-Cre- and hGFAP-Cre-dependent *Smo* KO mice exhibited smaller postnatal dentate gyruses and fewer proliferating cells than those in controls (Machold et al., 2003; Han et al., 2008). In *Kif3a* mutant and *Ift88* mutant mice, neuronal proliferation in the subgranular zone (SGZ) and subventricular zone (SVZ) was decreased, and Gli1 expression was abolished (Han et al., 2008; Tong et al., 2014; Gazea et al., 2016) These demonstrate that Shh signaling is involved in the proliferation and survival of NSCs and NPCs. Hypomorphic *Ift88*-mutant mice, in which *Ift88* mRNA and protein are reduced by 70–80%, showed disorganization of the midbrain dopominergic neuron (mDA) progenitors domain, and the number of mDA neurons was severly reduced (Gazea et al., 2016). Considering that primary cilia regulate neurogenesis by mediating Shh signaling, defects in learning and memory and ID might be caused by decreased neurogenesis due to aberrant Shh signaling. Expanding on the above research, additional studies are needed to determine how defective neuronal cilia lead to learning and memory impairment and ID.

## Concluding Remarks

In this review, we briefly summarized the interaction between primary cilia and several signaling pathways, including Shh, Wnt, MTOR, and autophagy, all of which are essential to cellular homeostasis. Given that neurological disorders are typical symptoms of ciliopathies, the importance of primary cilia during brain development cannot be emphasized enough. At present, however, we are unable to fully understand the underlying mechanisms by which defective neuronal cilia contribute the pathogenesis of neurological defects in ciliopathies. Future studies of neuronal cilia in relation to signaling pathways may provide better understanding of the functions of neuronal cilia and may help to develop novel treatments for ciliopathies by targeting neuronal cilia-mediated signaling pathways. Compared with considerable studies on neuronal cilia, there is a lack of research on the role of primary cilia in glial cells, another major component of the brain. Glial cells, such astrocytes and oligodendrocytes, also possess a single primary cilium (Berbari et al., 2007; Bishop et al., 2007; Kasahara et al., 2014). Recently, studies reported defective ciliogenesis in glioblastoma (GBM), a high-grade astrocytic malignancy, and demonstrated the potential role of primary cilia on GBM progression (Moser et al., 2014; Hoang-Minh et al., 2016a,b; Loskutov et al., 2018). Further studies on the role of primary cilia in glial cells, as well as neurons, will advance our understanding of the role of primary cilia in brain development.

To help achieve a better understanding of human ciliopathies resulting from mutations in ciliary genes, CRISPR/Cas9 genome editing of identified mutations in human ciliopathies or 3D culture of brain organoids derived from pluripotent stem cells from ciliopathy patients can be used to generate more pathophysiologically relevant models for ciliopathy, thereby overcoming the gap between animal models and human subjects. Also, increasing studies on interactions between primary cilia and other signaling pathways may uncover a previously unknown role for cilia in the pathogenesis of human neurological diseases that are not considered classical ciliopathies, such as FMCD, brain cancer (medulloblastoma and GBM), and neurodegenerative diseases (Alzheimer’s disease and HD) (Sotthibundhu et al., 2009; Keryer et al., 2011; Eguether and Hahne, 2018). Future studies of these disorders may help us to further the molecular basis of human diseases characterized by defects in primary cilia.

## Author Contributions

SP, HJ, and JL conceived the idea and wrote the manuscript.

## Conflict of Interest Statement

JL is a co-founder of SoVarGen, Inc. that develops new diagnostics and therapeutics for brain disorders. The remaining authors declare that the research was conducted in the absence of any commercial or financial relationships that could be construed as a potential conflict of interest.
